# Family functioning and delinquency among Chinese adolescents: Mediating effects of positive behavior recognition according to the humanistic perspective

**DOI:** 10.3389/fpubh.2022.985936

**Published:** 2022-09-29

**Authors:** Xingli Wan, Shuming Ji, Min Liu, Binxue Hong, Wei Shi, Liang Du, Li Zhao

**Affiliations:** ^1^Nursing Department, West China Second University Hospital, Sichuan University, Chengdu, China; ^2^The Key Laboratory of Birth Defects and Related Diseases of Women and Children, Sichuan University, Ministry of Education, Chengdu, China; ^3^Center of Biostatistics, Design, Measurement and Evaluation (CBDME), West China Hospital, Sichuan University, Chengdu, China; ^4^Department of Health Behavior and Social Medicine, West China School of Public Health and West China Fourth Hospital, Sichuan University, Chengdu, China; ^5^Department of Health Policy and Management, West China School of Public Health/West China Fourth Hospital, Sichuan University, Chengdu, China; ^6^Institute for Disaster Management and Reconstruction, Sichuan University, Chengdu, China; ^7^Chinese Evidence-Based Medicine Center, West China Hospital, Sichuan University, Chengdu, China; ^8^Department of Health Policy and Management, West China School of Public Health and West China Fourth Hospital, Sichuan University, Chengdu, China

**Keywords:** Chinese adolescents, delinquency, family functioning, humanistic perspective, mediator, positive behavior

## Abstract

**Background:**

Empirical research on the relationship between family functioning and delinquency has been sparse, although many studies have focused on the influence of family functioning on adolescent development. The current research aimed to fill this gap by exploring the influences of family functioning on adolescent delinquency and the mechanisms connecting the processes.

**Methods:**

We derived the baseline data from a prospective observational school-based cohort Chengdu Positive Child Development (CPCD) project. Students responded to a questionnaire containing validated measures of family functioning, positive behavior recognition, and delinquent behavior. We utilized structural equation modeling and maximum likelihood estimation to test the relationships.

**Results:**

Across 8811 Chinese adolescents, the incidence of delinquency behaviors among Chinese adolescents was relatively low. Family functioning and positive behavior recognition negatively predict delinquency (*p* < 0.001). Further, positive behavior recognition partially mediated the influence of family functioning on delinquency [*p* < 0.001, std. error = 0.01, 95% CI = (0.04, 0.07)]. Adolescents with better family functioning had little delinquency behavior, with positive behavior recognition and delinquency behavior negatively reinforcing each other.

**Conclusions:**

This study demonstrated that family functioning was a protective factor against adolescent delinquency and revealed that positive behavior recognition was a critical mediating mechanism linking family functioning to delinquency.

## Introduction

Behavioral problems refer to abnormal behaviors that exceed the normal range for the corresponding age in terms of severity and duration (1). Delinquency is a behavioral problem defined as engaging in antisocial behavior (2). Adolescence is a critical period of life development and a transition period characterized by physical, psychological, and social changes when adolescents learn to live independently (3). If adolescents cannot adequately cope with these developmental challenges, problematic behaviors such as delinquency likely emerge (4). Abundant evidence has revealed that delinquency is a growing global concern due to its high incidence, especially among early adolescents in both Western and Chinese backgrounds (5). For example, a representative sample study (*N* = 4,0502) showed that 46% of adolescents in Grade 7 to 12 in the United States engaged in offending behaviors (5). The studies conducted in Asia, such as in China and South Korea, have shown a relatively low but still significant prevalence of adolescent delinquency (6, 7). A large cross-sectional study reported that the total detection rate of behavioral problems in two districts in Beijing was 16.7% (8). A statistical report on China's Youth Development (2020) jointly released by the China Youth and Children Research Center and the International Liaison Department of the Central Committee of the Communist Youth showed that the number of criminals under 18 is rising (9).

Delinquency has been associated not only with poor outcomes such as alcohol and drug abuse and mental health problems (10–12) but also linked to long-lasting consequences (13), including severely hindering interpersonal development and even social sustainability (14, 15). Early and persistent delinquent acts strongly predicted violent behavior, partner conflict, unemployment later in adolescence (16, 17), and chronic antisocial behavior (18). Given its high prevalence and long-term disruptive outcomes, adolescent delinquency undoubtedly has become a severe social problem that has brought stress and high costs to families and society (19). Thus, identifying the predictive and protective factors for delinquency becomes an essential task of youth studies that could contribute to adolescent health programs.

The family environment is vital to the individual's psychological behavior (20). Family functioning refers to a model in which family members can obtain the necessary material and spiritual conditions to advance and promote their physical, mental, and social development in a healthy and beneficial direction (21). Although many theories and studies (22, 23) suggest that when an individual enters adolescence, his/her focus shifts from family and parents to peers, numerous studies have found that family still profoundly influences adolescents (24, 25). Bronfenbrenner proposes five environmental systems based on the ecosystem theory (1979), from the most private to the most general environment: microsystem, mesoscopic system, external system, macrosystem, and temporal system. Family and peers are the critical microsystem variables that influence an adolescent's risk-taking behavior (26). Family functioning is critical in evaluating the family environment, including role assignment, communication, emotional response and intervention, effective problem-solving ability, and behavior control (27). Psychologists and sociologists stress the importance of parental and positive parent-child relationships for children's growth and development (28, 29). Good family function includes high levels of love and support, positive communication, and behavioral discipline (30), providing positive emotional experience and cognitive attitude for individuals, promoting individual mental health (31) and good adaptation (32), and reducing the incidence of problem behavior (26). However, poor family functioning, such as parent-child conflict and marital conflict between parents, damages adolescents' psychological resources and leads to various problem behaviors (33). Although many studies have explored the influence of family functioning on adolescent development, few scholars in China, except Shek (4, 34, 35), have explored the protective effect of family function on adolescent delinquency and explained the negative correlation with delinquency from the dimension of family functioning. Based on this discussion, we proposed Hypothesis 1: Family function can negatively predict delinquency in Chinese adolescents.

Positive behavior includes visible skills that increase the likelihood of success and personal satisfaction in normative academic, work, social, recreational, community, and family settings. The term “positive behavior” can be seen as equivalent to “prosocial behavior” (36). Positive behavior recognition is the appropriate response of the social environment to such behavior (37). The goal of recognition is to encourage adolescents to continue exhibiting positive behavior. Some researchers have proposed that positive behavior recognition is related to the positive self-perception of adolescents and can increase their likelihood of positive and prosocial behavior (36). Therefore, positive behavior recognition is an essential internal psychological resource for adolescent development. Studies have shown that a high level of identification can effectively reduce adolescent Internet addiction, emotional disorders, and other problem behaviors (38). However, few studies have explored the relationship between positive behavior recognition and delinquency. Thus, we hypothesized that positive behavior recognition significantly and negatively predicts delinquency (Hypothesis 2).

Some studies have confirmed that good family function can positively predict adolescent positive behavior recognition (38–40). A study of 148 adolescents living in the Netherlands with an average age of 15 showed that good maternal communication facilitates adolescents' search for positive behavior identification (40). However, few studies have explored the association between family functioning and positive behavior recognition among Chinese adolescents. Therefore, we hypothesized that family functioning positively predicts Chinese adolescents' positive behavior recognition (Hypothesis 3).

According to the humanistic view, positive behavior signifies positive human development. Rogers, a key figure in humanistic psychology, proposed a theory closely related to positive behavior recognition. According to Rogers (41), unconditional positive respect is one condition that leads to healthy relationships. Unconditional positive respect is when one maintains an unconditional, positive attitude toward others that gives the recipient a sense of self-worth. Positive behavior identification through personal warmth can be an aspect of positive respect. In addition, a significant other's recognition of a child's or adolescent's good deeds through warm and supportive verbal and non-verbal gestures can kickstart an individual's internal organizational enhancement, turning the individual into a better person in the whole process. In this way, unconditional positive respect is critical to human functioning as a form of positive behavior recognition.

There is research on family functioning, positive behavior recognition, and delinquency behaviors. Although some studies have found links between marital discord, parenting, parental attachment, and adolescent delinquency, the underlying mechanisms behind these associations are unclear (42). The family environment has an important influence on individual psychological behavior. Because positive behavior recognition has been considered an essential personal protective resource, few researchers have examined the relationship between family functioning, positive behavior recognition, and delinquency. They rarely discussed the mediating role of positive behavior recognition from a humanistic perspective. Thus, we formulated our hypothesis: Positive behavior recognition is a mediator in the influence of family functioning on adolescent delinquency (Hypothesis 4).

Although there have been many empirical studies on the relationship between family function and adolescent delinquency in the West, whether the conclusions of this study could explain part of the family factors affecting juvenile delinquency in China is not certain. Although many researchers in China have realized that family functioning plays a vital role in influencing adolescent delinquency, few empirical studies have examined the relationship between family functioning and adolescent delinquency. This study aimed to fill the knowledge gap. Based on Rogers' Humanistic Perspective, we predicted that teenagers in a good family functioning environment might have a high level of concern and support, good communication and behavior, discipline, and unconditional positive respect. Therefore, their good deeds are more likely to be identified to enhance the internal organization, recognize positive behavior, increase prosocial behavior, and reduce delinquency.

Some studies on the prevention of adolescent problems suggest that prevention should focus on reducing and preventing problems, but this is a “pathological” approach and limits the public's understanding of the personal potential of adolescents (43). Adolescents are not “troubles” but valuable resources with abilities, potentials, and strengths that can be cultivated and utilized to promote their overall development and active functioning (44). Therefore, the present study aimed to identify and add another internal personal resource to protect adolescents from problem behaviors, hoping to contribute to developing adolescent health programs. However, other problems exist, such as the single research method, small sample size, and lack of high reliability and validity measurement tools. Moreover, the family factors influencing adolescent delinquency are very complex. Based on the humanistic perspective, we hope our study can provide Chinese data by carrying out a large sample empirical study in the context of Chinese culture.

**Figure 1:**
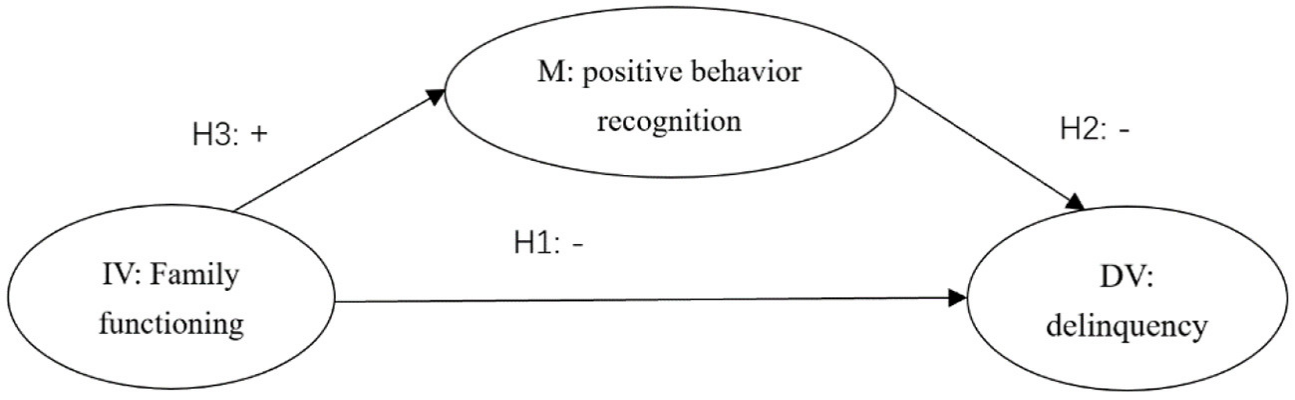
Hypothesized association among family functioning attributes, positive behavior recognition, and adolescent delinquency. IV, independent variable; M, mediator; DV, dependent variable.

Based on the above four assumptions, the present study proposed a mediation model as the conceptual framework of the present study (see Figure 1). A previous study has found that age and gender were associated with family functioning and adolescent delinquency (45). Hence, we included these demographic variables as control variables in this study.

## Materials and methods

### Participants

We derived the baseline data from a prospective observational school-based cohort Chengdu Positive Child Development (CPCD) Project, launched in December 2019 in Chengdu, the capital of Sichuan province. The CPCD Project aimed to investigate the current state of positive development and psychosocial and behavioral problems in children. The researchers adopted cluster and convenience sampling methods to select five primary and middle schools: one downtown, two in the suburbs in the south, and two in the north of Chengdu. All data are available (see Data Availability statement). The student's legal guardians gave written informed consent prior to participation. The informed consent included a project overview, survey procedures, potential benefits and risks, and a confidentiality agreement. The Medical Ethics Committee approved the study protocol of Sichuan University (K2020025).

### Investigative procedures

The project sent the questionnaires as the baseline survey. Students in Grades 1–9 who attended school on the survey date filled in the questionnaire.

Collection and non-response bias are two common types of bias in cross-sectional surveys. Some studies showed no significant difference between the paper-based and online collection methods (46). In addition, self-reported data may be susceptible to social desirability bias as the respondents may tend to provide answers perceived as more socially acceptable. A self-administered questionnaire could reduce the social-desirability bias (47) if the survey questions were sensitive. A lower response rate can easily cause researchers to worry about non-response bias. Transparent and organized paper-based questionnaire items could help participants answer questions quickly to reduce low responses.

Therefore, considering the feasibility of investigating primary schools, we took the following measures to ensure the authenticity and completeness of the information the students filled in.

First, we devised a paper-based and self-administered questionnaire with transparent and organized survey items. Second, we created a quiet and comfortable environment and provided the students plenty of time (40~60 min), and no one was allowed to submit in advance. All students independently completed the questionnaire under the supervision of the head teacher and investigators. Before the study began, the students could ask trained teachers questions when they had problems rather than discuss them with each other. Third, we used some negative scoring items in the questionnaire for logical checking. Finally, we collected the questionnaires on the spot, reviewed them, and promptly corrected them if there were any problems, such as missing data. Questionnaires with logical errors were excluded (For example, a questionnaire in which the respondents offered obvious contradictory answers was excluded).

### Measures

#### Assessment of family functioning

The Chinese Family Assessment Instrument (C-FAI) is a 33-item self-report scale developed to assess family functioning (48). The C-FAI has five subscales, including mutuality (mutual support, love, and concern among family members) (12 items), communication (frequency and nature of interaction among family members) (9 items), conflict and harmony (conflicting and harmonious behavior in the family) (6 items), parental concern (parental support behavior) (3 items), and parental control (harshness of parenting behavior) (3 items). Items include statements such as “Parents often talk to their children,” “Family members care about each other,” and “There is a lack of harmony among family members.” For each item, there is a five-point Likert scale (1 = very similar; 2 = somewhat similar; 3 = between somewhat similar and somewhat dissimilar; 4 = somewhat dissimilar; 5 = very dissimilar). Higher scores reflect greater dysfunction within the family. A previous study has confirmed that the C-FAI was a valid and reliable measure of family functioning (Cronbach's α = 0.933) (49). Our study also showed good reliability in delinquency (Cronbach's α = 0.936).

#### Assessment of delinquency

We used a 12-item 7-point scale to assess the occurrence of delinquent behaviors of the participants in the past year (50). For each item, there is a six-point scale (0 = never, 1 = one to two times; 2 = three to four times; 3 = five to six times; 4 = seven to eight times; 5 = nine to ten times; 6 = more than ten times). For complete scale information, please refer to Appendix A. A higher score on this scale denoted a greater degree of delinquency. This scale demonstrated good validity and reliability in the previous two studies (α = 0.76, α = 0.88, respectively) (50, 51). Cronbach's alpha in this study was 0.835.

#### Assessment of positive behavior recognition

We selected the Recognition for Positive Behavior subscale (PB) (51) from the Chinese Positive Youth Development Scale (CPYDS) designed by Shek et al. (5). There are four items in the subscale. The items were developed after reviewing the literature (52) and discussing between the first two authors. For each item, there is a six-point scale (1 = strongly disagree; 2 = relatively disagree; 3 = slightly disagree; 4 = slightly agree; 5 = relatively agree; 6 = strongly agree). For complete scale information, please refer to Appendix B. Higher scores on this scale indicated greater positive behavior recognition. The previous study has shown the scale had good validity and reliability (α = 0.76 and 0.80 at pre-and post-test) (53). Cronbach's α for this scale in our study was good (α = 0.772).

### Data analysis

We used the EpiData 3.1 (EpiData Association, Odense, Denmark) for double data entry and logic error verification. We used student's *t*-test to compare continuous variables (age, body mass index [BMI]) and the Mann–Whitney U test to compare non-normally distributed continuous variables (family functioning, positive behavior recognition, delinquency). To compare categorical variables (sex), we used the Chi-square test. Any *P*-values <0.05 were considered statistically significant.

To test the hypothetical models depicted in Figure 1, we performed the structural equation model in R version 4.1.3 with packages lavaan and semplot, using maximum likelihood estimation with robust standard errors. We performed the structural equation modeling analysis in two steps: (i) We examined the total effect of family functioning on positive behavior recognition and delinquency; (ii) We examined the direct and indirect effects of family functioning on delinquency, considering positive behavior recognition as a potential mediator. We considered that the adolescents' positive behavior recognition might display diversity due to their different ages, sex, and BMI. Therefore, age, sex, and BMI were included in the model to correct these effects on adolescents' positive behavior recognition.

In order to facilitate the interpretation of model results and the comparison of effect values, we multiplied the score of C-FAI by −1 to keep all correlations in the same direction. In addition, we assessed the overall fitting performance of the model by using the Comparative Fit Index (CFI), normed fit index (NFI), non-normed fit index (NNFI), and root mean square error of approximation (RMSEA) and other indicators. Generally speaking, CFI, NFI, and NNFI values >0.9 and RMSEA values <0.08 are considered good model fit indices (54). However, the cut-off value of the structural equation model fit indices is always controversial. MacCallum et al. proposed that an RMSEA of between 0.08 to 0.10 provides a mediocre fit and below 0.08 shows a good fit (55). The cut-off as low as 0.80 has been proferred; however, Bentler and Hu have suggested NNFI ≥ 0.95 as the threshold (56).

## Results

### Descriptive statistics

We sent out a total of 8,968 questionnaires during the baseline survey, and a total of 8,811 students in Grades 1–9 (aged 6–16) who attended school on the survey date have completed the questionnaire, with a response rate of 98.4%. There were slightly more boys than girls (51.6 vs. 48.4%) and more primary than middle school students (62.7 vs. 37.3%). More students lived in urban than rural areas (65.0 vs. 35.0%). Moreover, the average age of boys and girls is 12.86 ± 2.33 and 12.90 ± 2.32, respectively.

### The detection rate of delinquency behavior

Results in Table 1 show that delinquency behaviors were relatively low among Chinese adolescents. More than 90% of the respondents reported that they had never stolen, fought in a gang, or had a sexual relationship with others (other than assault and damaging others' properties) in the past year. In contrast, about half of the respondents reported having cheated on somebody or spoken foul language.

**Table 1:** Summary table of the reported frequency of delinquency behavior (*N* = 8,811).

**Delinquency**	**Never**	**1–2 times**	**3–4 times**	**5–6 times**	**7–8 times**	**9–10 times**	**>10 times**	**Mean**
	**(%)**	**(%)**	**(%)**	**(%)**	**(%)**	**(%)**	**(%)**	**times**
Stealing	91.38	6.56	1.16	0.19	0.14	0.80	0.50	0.13
Cheating	57.82	26.75	8.73	2.742	0.86	0.49	2.61	0.74
Truancy	96.57	1.99	0.61	0.30	0.17	0.03	0.33	0.07
Running away from home	93.12	4.81	0.90	0.53	0.15	0.09	0.41	0.12
Damaging others' property	87.72	9.24	1.65	0.59	0.24	0.11	0.45	0.19
Assault	87.15	8.91	1.80	0.79	0.32	0.23	0.81	0.22
Having a sexual relationship with others	93.81	3.94	0.87	0.48	0.26	0.16	0.48	0.12
Gang fighting	94.07	3.77	0.87	0.42	0.19	0.20	0.47	0.11
Using foul language	46.43	28.28	9.27	4.42	1.80	0.96	8.85	1.25
Staying away from home without parental consent	94.88	3.00	0.91	0.42	0.24	0.08	0.48	0.10
Strong-arming others	90.26	6.41	1.46	0.70	0.31	0.22	0.65	0.18
Breaking into residences	95.80	2.65	0.53	0.31	0.14	0.20	0.37	0.08

### Descriptive statistics of the participants on delinquency, family functioning, positive behavior recognition

Table 2 indicates boys scored significantly higher on delinquency than girls (*t* = 11.24, *P* < 0.001). Boys also scored higher on family functioning than girls (*t* = 3.44, *P* < 0.001), indicating boys have higher family dysfunction levels than girls. However, boys scored lower on positive behavior recognition than girls (*t* = −3.79, *P* < 0.001).

**Table 2:** Descriptive statistics of the participants on delinquency, family functioning, and positive behavior recognition.

**Variables**	**Boys (*N =* 4,543)**					**Girls (*N =* 4,268)**						
	**Min**	**Max**	**Mean**	**Variance**	**SD**	**Min**	**Max**	**Mean**	**Variance**	**SD**	**Test value**	***P*-value**
Family functioning	31	155	56	549.95	23.45	31	155	35	522.26	22.85	3.44	<0.001
Mutuality	6	30	10.68	28.53	5.34	6	30	10.48	26.39	5.14	1.84	0.07
Communication	5	25	9.61	27.38	5.23	5	25	9.76	26.38	5.14	−1.27	0.20
Conflict and harmony	10	50	20.07	70.68	8.41	10	50	19.55	65.97	8.12	2.96	<0.01
Parental concern	7	35	13.23	46.82	6.84	7	35	12.62	39.74	6.3	4.34	<0.001
Parental control	3	15	6.91	13.08	3.62	3	15	6.41	11.58	3.4	6.73	<0.001
Positive behavior recognition	4	24	20.03	16.51	4.06	4	24	20.35	13.58	3.69	−3.79	<0.001
Delinquency	0	72	3.98	50.63	7.12	0	59	2.59	17.64	4.2	11.24	<0.001

### Correlations among variables

Table 3 shows the correlations among variables. Family functioning was negatively related to delinquency (*r* = −0.28, *P* < 0.001). The four dimensions of Family Functioning (mutuality, communication, conflict and harmony, and parent concern) had a relatively higher correlation (*r* = −0.25, *r* = −0.25, *r* = −0.24, *r* = −0.23, *P* < 0.001, respectively) to delinquency than parent control (*r* = −0.13, *P* < 0.001). Positive behavior recognition was negatively related to delinquency (*r* = −0.21, *P* < 0.001). Family functioning was positively correlated with positive behavior recognition (*r* = 0.34, *P* < 0.001). Overall, these observed results are consistent with our original hypotheses.

**Table 3:** Intercorrelation matrix for family functioning variables, positive behavior recognition, and delinquency.

**Variables**	**1**	**2**	**3**	**4**	**5**	**6**	**7**	**8**	**9**	**10**	**11**
1. Family Functioning	—										
2. Mutuality	0.85^**^	—									
3. Communication	0.80^**^	0.76^**^	—								
4. Conflict and harmony	0.90^**^	0.67^**^	0.60^**^	—							
5. Parent concern	0.84^**^	0.59^**^	0.55^**^	0.72^**^	—						
6. Parent control	0.46^**^	0.30^**^	0.20^**^	0.32^**^	0.28^**^	—					
7. Recognition of positive behavior	0.34^**^	0.29^**^	0.26^**^	0.30^**^	0.28^**^	0.21^**^	—				
8. Delinquency	−0.28^**^	−0.25^**^	−0.25^**^	−0.24^**^	−0.23^**^	−0.13^**^	−0.21^**^	—			
9. Gender	0.04^*^	0.02	−0.01	0.03^*^	0.05^**^	0.07^**^	0.04^*^	−0.12^**^	—		
10. Age	−0.01	0.01	−0.03	−0.02	−0.03^*^	0.07^**^	−0.17^**^	0.13^**^	0.01	—	
11. BMI	−0.01	0.01	−0.01	−0.01	−0.01	0.03	−0.08^**^	0.07^**^	−0.02	0.44^**^	—

** p < 0.001,

* p < 0.05. Minor correlation when 0 ≤ R < 0.2, slight correlation for 0.2 ≤ R < 0.4, moderate correlation for 0.4 ≤ R < 0.7, and high correlation for 0.7 ≤ R ≤ 1.0.

### Predictions of delinquency and mediating effect of positive behavior recognition

#### Model testing

The model assessment showed an acceptable model fit. The results were χ^2^ = 2,533.635, df = 31, CFI =0.896, NFI = 0.895, NNFI = 0.859, RMSEA = 0.096 [95% CI = (0.093, 0.096)]. More information about the Model fit is in Appendix C.

#### Testing of mediation effects

**Figure 2:**
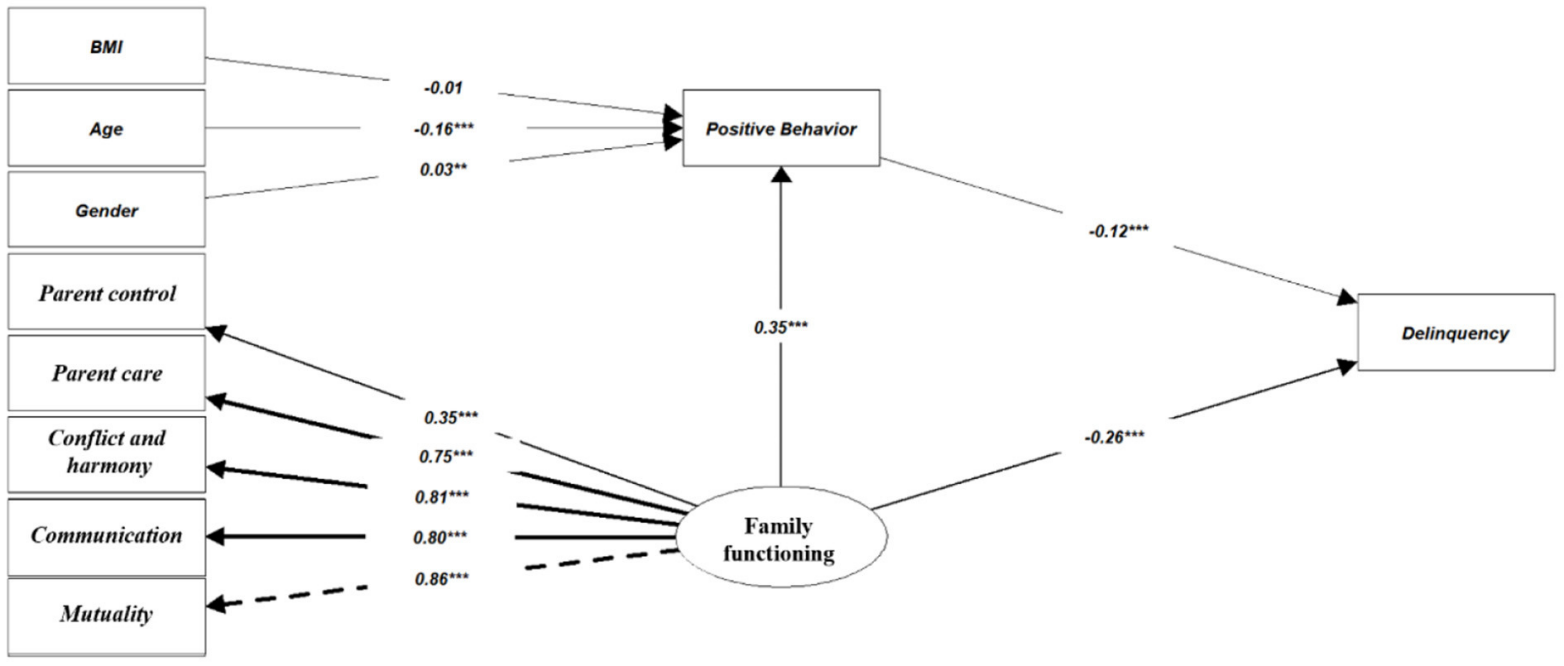
Standardized path coefficients for the relationship between family functioning, positive behavior recognition, and delinquency. ^***^p < 0.001, ^**^p < 0.01.

Figure 2 outlines the standardized path coefficients and path significance of the relationships between family functioning, positive behavior recognition, and delinquency. First, family functioning (β = −0.26, *P* < 0.001) negatively predicted adolescent delinquency, supporting Hypothesis 1.

Second, positive behavior recognition showed negative predictive effects on adolescent delinquency (β = −0.12, *P* < 0.001), supporting Hypothesis 2.

Third, Family functioning positively contributed to positive behavior recognition (β = 0.35, *P* < 0.001), supporting Hypothesis 3.

Table 4 presents the path coefficients and the decomposition of effects in the structural mediation model. When using positive behavior recognitions as the mediator, the indirect effects of family functioning on delinquency were also significant, supporting the mediating effect model [β = −0.04, b = −0.06, 95% CI = (−0.07, −0.04), *P* = 0]. Overall, the mediation model explained 10.1% of the variance in delinquency.

**Table 4:** Presents the path coefficients and indirect effects of family functioning on delinquency.

**Variables**	**Standardized regression coefficient**	**beta**	**Std. error**	***z***	***P*-value**	**95% CI lower**	**95% CI upper**	***R* ^2^**	***f* ^2^**
**Measurement model**									
Family functioning (FF) → Delinquency	−0.26	−0.34	0.03	−12.94	0	−0.39	−0.29		
FF → Positive behavior recognition (PB)	0.35	0.30	0.01	25.28	0	0.28	0.32	0.147	0.172
PB → Delinquency	−0.12	−0.19	0.02	−7.63	0	−0.23	−0.14		
**Structural model**									
Direct FF → Delinquency	−0.26	−0.34	0.03	−12.94	0	−0.39	−0.29		
Direct PB → Delinquency	−0.12	−0.19	0.02	−7.63	0	−0.23	−0.14		
Indirect FF → PB → Delinquency	−0.04	−0.06	0.01	−7.37	0	−0.07	−0.04		
Total FF → PB → Delinquency	−0.42	−0.58	0.03	−20.25	0	−0.63	−0.52	0.101	0.112

## Discussion

### Family functioning negatively predicts adolescent delinquency

Regarding our first research question, correlation and regression analyses consistently revealed that family functioning could negatively predict adolescent delinquency. Adolescents with better family mutuality, communication, parental concern, and fewer family conflicts had less delinquency behavior. These findings supported our initial hypothesis 1. These results are also consistent with the findings of other studies (34, 35, 57, 58). A positive and supportive family environment could reduce delinquent behavior because children who received warm care from their parents were more likely to conform to rules and develop positive behavior (59, 60).

As adolescents grow up, they relate to non-family members such as their peers (61); more family conflicts and poor family communication might even push them to seek non-familial support (57). The difficulties children have experienced at home are then carried over into school life and peer relationships, resulting in disrupted relationships with mainstream peer groups. To regain a sense of membership, those individuals whom conventional peers reject may join delinquent peer groups and have their antisocial behaviors reinforced (35). Previous studies have shown that parental control and concern (parental support behavior) effectively reduce adolescent conduct problems (62–65). Authoritative parenting is conducive to establishing a positive parent-child relationship, facilitating children's open communication with parents, enhancing parents' ability to identify potential risks children may encounter, and intervening when necessary (66). However, the correlation between parent control and delinquency in the present study was relatively low compared to the other four dimensions of family functioning, as shown in Table 3. This may suggesting that parental control is less important than the other four factors, further strengthening other four aspects of family functioning may increase the extent to which family functioning contributes to adolescent delinquency.

In general, the findings are congruent with the family systems theory expectation that the development of family function attributes provides the essential external resources to protect adolescents from delinquency. The current findings broaden existing family function literature by including an Asian sample, further extending the conclusion to China mainland, suggesting that the general negative relationship between family functioning and delinquency may hold for different cultural contexts.

### Positive behavior recognition serves as a mediating factor

Consistent with previous studies, our findings showed that family functioning was positively related to positive behavior recognition, and both were inversely associated with delinquency. These findings also supported Hypotheses 2 and 3. These results are consistent with those of other studies (38–40, 43, 67–69).

For the fourth research question, the overall findings also support our hypothesis that positive behavior recognition is a mediator predicting the effect of family functioning on delinquency.

According to the humanistic theory, human behavior and experiences are guided by one essential striving in life: the fundamental tendency to develop all capacities to enhance the person's functioning, thereby generating positive behavior (70). All urges, desires, wants, goals, values, and motives are subsumed under “organismic enhancement.” A person will become all that he or she can become with his or her potential fulfilled. According to Rogers, one interpersonal condition that leads to healthy development is unconditional positive regard, which is essential to healthy development. The absence of this condition may lead people to view themselves negatively. Human services professionals believe that giving people unconditional positive regard and acceptance provides the best possible conditions for personal growth (41). Good family functions, such as the harmonious relationship between parents, harmonious communication between parents and children, and family cohesion, are critical for adolescents to gain positive identity and unconditional acceptance (71).

Based on Rogers' Humanistic Perspective, adolescents in a well-functioning family environment receive a high level of parental concern and support, maintain good family mutuality, communication, and behavioral discipline, and are more likely to receive unconditional positive respect. Therefore, their good deeds are more likely to be recognized, activating their internal organizational enhancement and making positive behavioral identifications to increase prosocial behavior and reduce delinquency.

Overall, the findings suggest that family functioning enables adolescents to present more positive behavior recognition, leading to a lower delinquency level.

Based on the current results, cultivating inner strengths (such as positive behavior recognition) for Chinese adolescents is a promising strategy to promote adolescents' well-being and protect them from delinquency. In addition to adopting positive behavior recognition through good family functioning, instructors are also encouraged to inject the meaning of positive behavior during various activities throughout the program implementation in primary schools. Adolescents are an essential part of the urban population, and their physical and mental health is also the focus of the government, school, and family. This study also provides data on the adolescent population for urban population health research.

### Strengths and limitations

This study adds to the few related studies in this area, particularly in the Chinese context. It is the first study to add theoretical and practical value to the existing literature by deepening our understanding of the mediating role of positive behavior recognition from a humanistic perspective. A second strength is that the present study also attempts to identify internal personal resources to protect adolescents from problem behaviors that would contribute to developing adolescent health programs. A third strength is that there are few related studies in Chinese contexts, so we recruited Chinese adolescents for the present study. A final strength is the large sample size.

A limitation of this study is the cross-sectional design. In order to conclude the causal relationship between the study variables, further prospective studies are needed. Second, as the present model is based on a large sample collected in Chengdu, mainland Chinese, the generalizability of the findings to adolescents in other Chinese cities remains unknown at this stage. Therefore, future studies should replicate these results to test our findings' generalizability. Third, the model fit is acceptable in the present study. However, there are specific percentages of unexplained variance in the model, which indicates that other potential variables should be included in the model to give a higher degree of prediction. Focus on other inner personal resources could be the future research topic.

Fourth, we collected the data through students' self-reporting, which might increase the likelihood of reporting bias. However, we chose self-administrated paper surveys and collected the questionnaires on the spot, which reduced this problem. In addition to including the reporting data from parents and teachers, future research could consider using methodological approaches, such as multidimensional item response theory and responder misclassification correction formulas (72, 73), to analyze the self-reported outcome bias.

## Conclusions

Our survey methodology was feasible, understandable, and helpful in providing data on the prevalence of delinquency in Chinese adolescents. This study demonstrated that family functioning was a protective factor against adolescent delinquency and revealed that positive behavior recognition was a critical mediating mechanism linking family functioning to delinquency. The present findings represent a significant advance in the literature on family functioning and delinquency, particularly in the Chinese context.

## Data availability statement

The raw data supporting the conclusions of this article will be made available by the authors, without undue reservation.

## Ethics statement

The study protocol was approved by the Medical Ethics Committee of Sichuan University (K2020025). Written informed consent to participate in this study was provided by the participants' legal guardian/next of kin.

## Author contributions

Conceptualization: XLW and LD. Formal analysis and data curation: SMJ. Investigation: ML and BXH. Funding acquisition, project administration, resources, and supervision: LZ. Writing—original draft: XLW. Writing—review and editing: WS and LD. All authors have read and agreed to the published version of the manuscript.

## Funding

This study was funded by the Study of Diet and Nutrition Assessment and Intervention Technology from Active Health and Aging Technologic Solutions Major Project of National Key R&D Program, Grant Number 2020YFC2006300 and the International Institute of Spatial Lifecourse Epidemiology and Hong Kong Polytechnic University, Grant Number 19H0642.

## Conflict of interest

The authors declare that the research was conducted in the absence of any commercial or financial relationships that could be construed as a potential conflict of interest.

## Publisher's note

All claims expressed in this article are solely those of the authors and do not necessarily represent those of their affiliated organizations, or those of the publisher, the editors and the reviewers. Any product that may be evaluated in this article, or claim that may be made by its manufacturer, is not guaranteed or endorsed by the publisher.
