# Supplementary material for: Family functioning and delinquency among Chinese adolescents: Mediating effects of positive behavior recognition according to the humanistic perspective
Source: Front Public Health. 2022 Sep 29;10:985936. doi: 10.3389/fpubh.2022.985936 (PMC9557932; doi:10.3389/fpubh.2022.985936)
Supplement: Supplementary file 1 [file Table_1.docx]

**Appendix A**

**Delinquency Scale Items**

| **Number** | **Items** |
| --- | --- |
| **1** | Stealing things |
| **2** | Cheating on examinations |
| **3** | Truancy |
| **4** | Running away from home |
| **5** | Damaging the property of other people |
| **6** | Attacking other people physically |
| **7** | Having sex with others |
| **8** | Fighting in gangs |
| **9** | Speaking foul language |
| **10** | Not returning home without parental permission |
| **11** | Strong-arming others |
| **12** | Breaking into residences |
