# Supplementary material for: Family functioning and delinquency among Chinese adolescents: Mediating effects of positive behavior recognition according to the humanistic perspective
Source: Front Public Health. 2022 Sep 29;10:985936. doi: 10.3389/fpubh.2022.985936 (PMC9557932; doi:10.3389/fpubh.2022.985936)
Supplement: Supplementary file 2 [file Table_2.docx]

**Appendix B**

**Positive Behavior Recognition Scale**

| **Number** | **Items** |
| --- | --- |
| 1 | My teachers would give me compliments when I had tried my best. |
| 2 | When I help others, my classmates will recognize my behavior. |
| 3 | At school, teachers value whether I do things well or not. |
| 4 | I think the teacher rewards and punishes students' behavior clearly. |
