# Supplementary material for: Family functioning and delinquency among Chinese adolescents: Mediating effects of positive behavior recognition according to the humanistic perspective
Source: Front Public Health. 2022 Sep 29;10:985936. doi: 10.3389/fpubh.2022.985936 (PMC9557932; doi:10.3389/fpubh.2022.985936)
Supplement: Supplementary file 3 [file Table_3.docx]

**Appendix C**

**Fitting indices of different estimators**

| **Estimators methods** | **NFI** | **NNFI** | **CFI** | **SRMR** | **RMSEA** | **RMSEA ci lower** | **RMSEA ci upper** |  |
| --- | --- | --- | --- | --- | --- | --- | --- | --- |
| ML | 0.895 | 0.859 | 0.896 | 0.041 | 0.096 | 0.093 | 0.099 |  |
| DWLS | 0.969 | 0.960 | 0.971 | 0.040 | 0.045 | 0.041 | 0.048 |  |
| ULS | 0.991 | 0.988 | 0.991 | 0.041 | Not applicable | | |  |
| WLS | 0.809 | 0.748 | 0.814 | 0.074 | 0.057 | 0.054 | 0.060 |  |

Notes: The cut-off value of the structural equation model fit indices is always controversial. MacCallum et al. proposed that an RMSEA of between 0.08 to 0.10 provides a mediocre fit and below 0.08 shows a good fit(56). The cut-off as low as 0.80 has been proferred; however, Bentler and Hu (1999) have suggested NNFI ≥ 0.95 as the threshold (57).
